# Application research on reducing radiation-induced lung injury with a trigger operator based on overlap volume histogram (OVH) in breast cancer postoperative radiotherapy

**DOI:** 10.1038/s41598-023-49282-z

**Published:** 2023-12-12

**Authors:** Qianyan Li, Feifei Deng, Xiang Pan, Han Bai, Jie Bai, Xuhong Liu, Feihu Chen, Ren Ge

**Affiliations:** 1 Department of Radiation Oncology, The Third Affiliated Hospital of Kunming Medical University, Yunnan Tumor Hospital, Kunming, Yunnan China; 2 Department of Oncology, 920Th Hospital of Joint Logistics Support Force, PLA, Kunming, Yunnan China; 3 Department of Physics and Astronomy, Yunnan University, Kunming, Yunnan China; 4 Department of Radiation Oncology, Daqin Tumor Hospital, Guiyang, Guizhou, China; 5 Department of Health Technology and Informatics, The Hong Kong Polytechnic University, Hongkong, China

**Keywords:** Biomedical engineering, Breast cancer

## Abstract

This study aims to develop a trigger operator based on the Overlap Volume Histogram (OVH) and examined its effectiveness in enhancing plan quality to minimize radiation-induced lung injury in postoperative radiotherapy for breast cancer. This trigger operator was applied for plan re-optimization to the previous Volumetric Modulated Arc Therapy (VMAT) plans of 16 left breast conserving surgery cases. These cases were categorized into a Contiguous Group (CG) and a Separated Group (SG) based on the relative position between the target and the Left-Lung (L-Lung). We investigated the changes in Vx, mean dose, and Normal Tissue Complication Probability (NTCP) values of organs-at-risk (OARs) before and after using the trigger operator. The Pairwise Sample T test was employed to evaluate the differences in indices between the two groups before and after optimizations. The trigger operator effectively initiated plan re-optimization. The values of V5, V10, V20, V30, and V40 of the L-Lung, as well as the mean dose of the heart, all decreased after re-optimization. The Pairwise Sample T test results showed statistically significant differences in the V20, V30, and V40 of the L-Lung in the CG (*P* < 0.01), and in the V5, V10, V20, V30, and V40 of the L-Lung in the SG (*P* < 0.01). Our findings suggest that the proposed trigger operator can improve plan quality, thereby reducing radiation-induced lung injury in postoperative radiotherapy for breast cancer.

## Introduction

Radiotherapy is one of the standard methods for cancer treatment. The rapid advancements in radiation physics and computer technology have significantly propelled the development of radiotherapy techniques. One primary aim of these advancements is to improve the treatment-gain-ratio. Intensity-Modulated Radiation Therapy (IMRT), with its robust dose adjustment capability, optimizes the dose concentration on the target volume while minimizing the dose to peripheral organs at risk (OARs). However, achieving this objective is often a labor-intensive process involving trial and error. Planners manually and repeatedly adjust the cost function until they obtain clinically acceptable Dose-Volume Histograms (DVH) and dose distributions. Consequently, the quality of the plan heavily relies on the planner's experience and the amount of time they can dedicate to the process.

The level of experience among planners is widely acknowledged to vary, leading to discrepancies in the quality of radiotherapy plans. Numerous studies have shown that centers with more experienced planners tend to produce higher quality IMRT plans compared to those with less experienced planners. Furthermore, even within the same center, the quality of plans can differ significantly depending on the individual planner's experience and expertise^1,2^. Because, for OARs, in addition to some criteria recommended by Radiation Therapy Oncology Group (RTOG) and the American Association of Physicists in Medicine (AAPM), etc., planners often lack a formal method for evaluating DVHs of OARs after the completion of a new plan. Although these criteria recommended by RTOG and AAPM are useful, they typically provide upper dose limits for OARs based on general population data^3,4^. For a specific plan, the lower dose limit that OARs may reach is more clinically significant than the upper dose limit. For instance, the RTOG standard sets an upper limit of V20 < 33.0% for pulmonary dose^5^, which is easily achievable in radiotherapy after breast conserving surgery^6^. One of the potential challenges in radiotherapy planning is mathematically defining the DVH objectives that balance the trade-offs between target coverage and sparing of normal tissues. The proposal and application of the Overlap Volume Histogram (OVH) have addressed these issues to a certain extent, providing a more nuanced approach to treatment planning^7^. The OVH provides a way to predict the likely DVHs of the OARs by comparing the relative spatial configurations against those of previous patients. In this method, a database of prior patients will be built, and new patients’ DVHs mainly depend on the DVHs quality of prior patients in the database, which is one defect in the OVH application. In addition, OVH is a curve graph, comparing curves from different patients is inconvenient. Because it is difficult to judge that two OVH curves are similar (close) except that they overlap completely, and in fact, there are no two patients whose OVH curves overlap perfectly. Therefore, our team attempted to introduce a trigger operator based on OVH to continually improve the quality of plans for breast cancer.

## Materials and methods

### Materials

We conducted a retrospective analysis of 16 patients who underwent left breast-conserving surgery using VMAT techniques. These plans were randomly selected from the clinical plans treated at our hospital. 16 cases were prescribed with 50 Gy in 25 fractions. The 6 MV photon energy and ELKTA VERSA HD linear accelerate machine with Monaco TPS (Version: 5.11.03) was utilized to optimize the plan. The calculation properties for this study were as follows: (1) Grid Spacing (cm): 0.3, (2) Calculate Dose Deposition to Medium, (3) the Algorithm is Monte Carlo Photon. For re-optimization, the gantry angle ranged from 150 to 270 degrees (counter-clockwise, CCW), with a tangent field and two arcs. Each arc had 120 control points, and the minimum segment width was 0.5 cm. Furthermore, the volume of the Planning Target Volume (PTV) and Left-Lung (L-Lung) for the 16 patients is presented in Table 1. After setting some optimizing constraints (shown in Table 2), the dose curve distribution was automatically optimized. Through repeated parameter adjustments, the ideal dose curve distribution was achieved. This study was conducted in accordance with the ethical and data security guidelines of the Ethics Committee of the Third Affiliated Hospital of Kunming Medical University, Yunnan Cancer Hospital, China. All research methods were approved by the same committee.

**Table 1:** The volume of PTV and L-Lung in the 16 plans in the CG and SG.

CG	Patient NO	1	2	3	4	6	7	8	12	13	14
	V(PTV)	623.22	876.70	426.75	1221.58	682.52	636.5	412.69	388.06	682.02	777.90
	V(L-Lung)	1278.51	813.54	1069.64	1224	1242.74	1363.84	1248.06	1152.55	1400.73	802.37
SG	Patient NO	5	9	10	11	15	16				
	V(PTV)	932.44	1615.03	488.47	444.81	1013.06	1088.01				
	V(L-Lung)	914.53	1122.03	1079.58	1431.65	930.06	1288.53				

*Patient NO is the number of 16 patients.

**Table 2:** The optimization constraints of plans.

PTV/OARs	Constraint conditions	Objectives
PTV	D99	≥ 50 Gy
	Dmax	≤ 55 Gy
L-Lung	V20	< 30%
R-Lung	V20	< 10%
Heart	Dmean	< 4 Gy
Contralateral Breast	Dmean	< 6 Gy
Cord	Dmax	< 30 Gy

### Methods

#### Definition of the OVH

**Figure 1:**
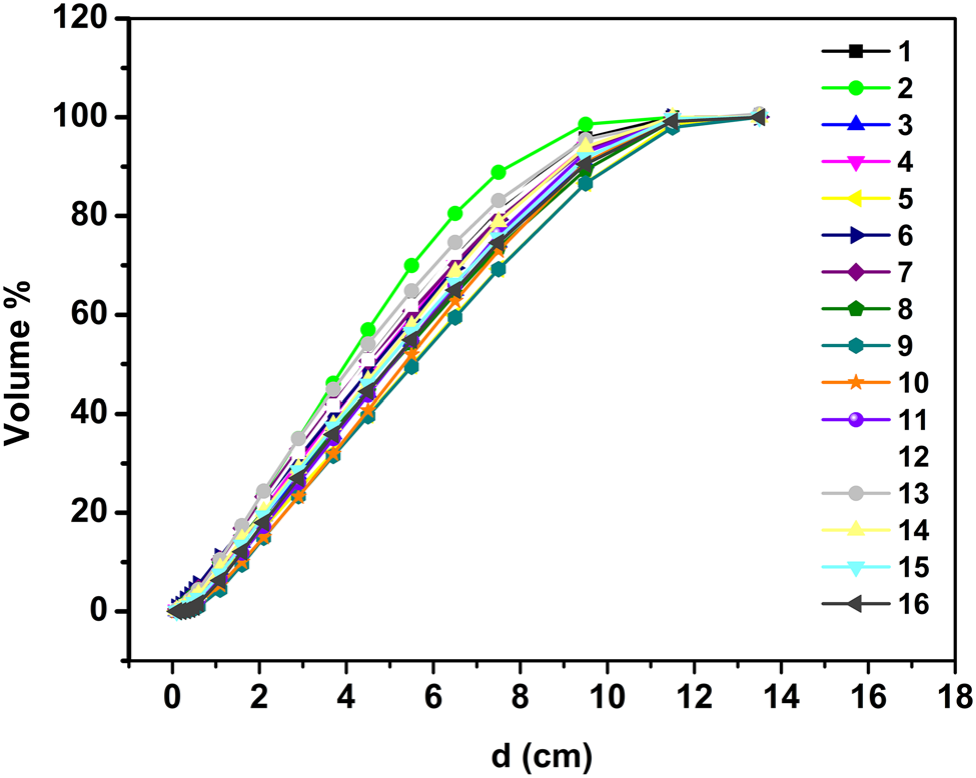
The L-Lung’s OVH curves of 16 patients.

In this study, a one-dimensional function, overlap volume histogram (OVH) *v* = OVH(*d*), was used to characterize the three-dimensional spatial relationship between an OAR and a target. The OVH was defined as follows: the value of the OVH represents the percentage of the OAR’s volume that overlaps with a uniformly expanded/or contracted target^8^.

1 $${\text{OVH}}\left( {\text{r}} \right){ = }\frac{{{\text{V}}\left\{ {{\text{P}} \in {\text{O|d(P, T)}} \le \left. {\text{r}} \right\}} \right.}}{{{\text{V}}\left( {\text{O, All}} \right)}}$$

where P is a subset of O, T is the tumor, O is the organ at risk (OAR); V (O, all) is the total volume of the OAR; d (P, T) is the distance from p to the boundary of the tumor; {P ∈ O|d(P,T) ≤ r} represents a subset of O whose distance to the tumor is less than r. According to the above OVH, the 16 VMAT plans from 16 patients after left breast conserving surgery were retrospectively analyzed. These plans were randomly selected from the clinical plans treated at our hospital. The OVH between PTV and selected OAR (L-Lung) was generated for each case. We first uniformly expanded the target in all directions by a distance of 'a' mm (including 6 pieces of 1 mm, 3 pieces of 5 mm, 3 pieces of 8 mm, 3 pieces of 10 mm, 3 pieces of 20 mm). We then calculated the overlap volume between the expanded target and the L-Lung. The expansion with 'a' mm was repeated until the expanded target fully encompassed the L-Lung, in which situation the overlap volume was the volume of the L-Lung. Plot the OVH curve (Fig. 1).

### Classification of OVH

#### A simple example describes the properties of OVH

**Figure 2:**
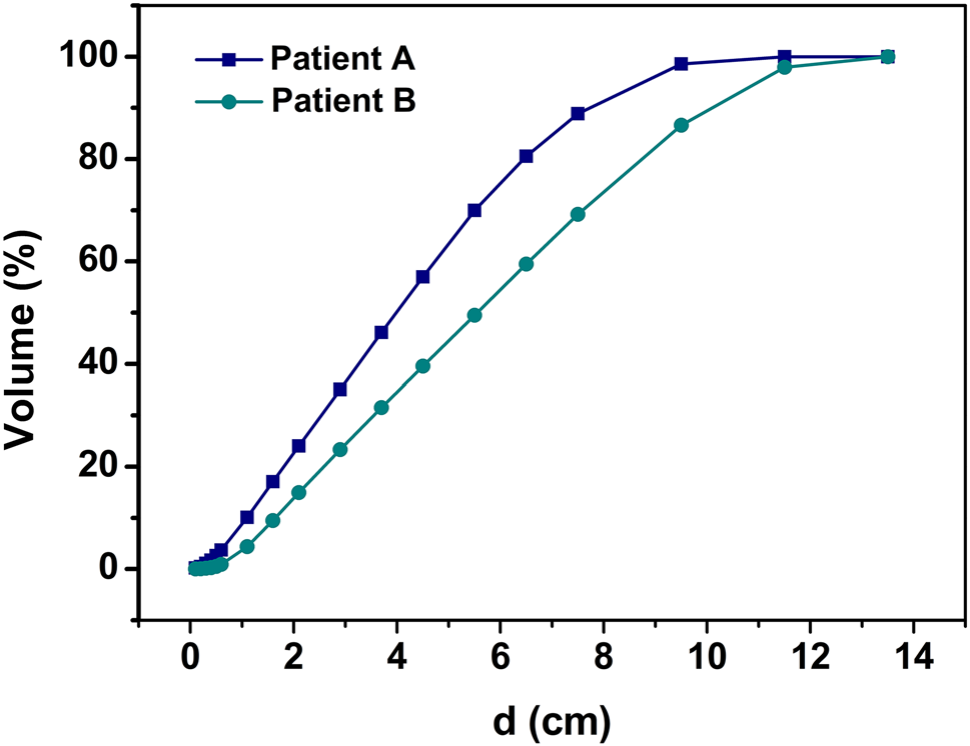
The L-Lung’s OVH curves of representative patient's A and B.

Figure 2 shows the OVH curves of representative patients A and B from 16 cases, it can be concluded by combining formula (1) and Fig. 2: theoretically, when v > OVH (0), patient B should receive a lower dose of L-Lung than patient A in any volume, we have *V*_*d*, Patient B_ < *V *_*d*_, _Patient A_, then D_*d*, Patient B <_ D_*d*, Patient A_, when v = OVH (0), Patient B's L-Lung receive the same dose as Patient A, we have *V *_*d*, Patient B_ = *V *_*d*_, _Patient A_, then D_*d*_, _Patient B_ = D_*d*, Patient A_. Obviously, the OVH curve could act as a guideline to optimize plans.

**Figure 3:**
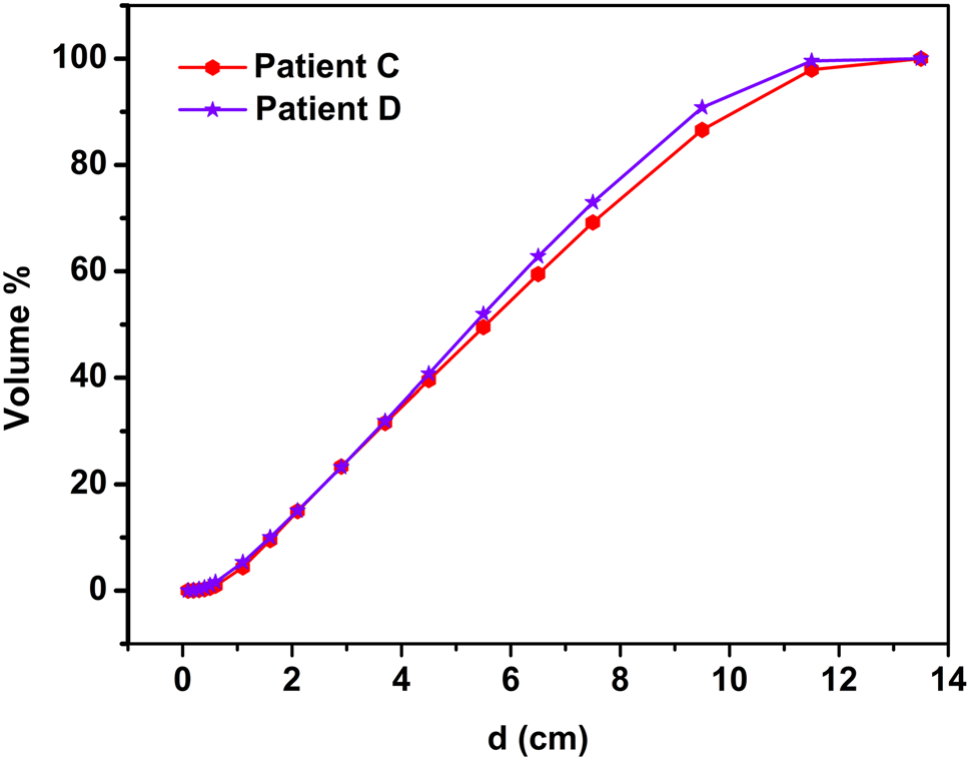
The L-Lung’s OVH curves of representative patient's C and D.

In addition, as shown in Fig. 3, the OVH curves of the two patients can invert when the PTV expansion distance reaches a certain value. At this point, plan optimization guided by the entire OVH curve may not fully align with the prediction. Since OVH is a one-dimensional function related to OARs, it offers a method to infer the potential DVH of OARs by comparing the relative spatial configurations between patients. However, the relationship between OARs and PTV is three-dimensional, and the spatial relationship between PTV and OARs can vary in multi-slice and single-slice CT scans. According to the literature^8^, the spatial relationship between PTV and OARs can be divided into three types: contiguous, separated, and intersecting.

OVH was a function of distance d, OVH = *f* (d), but in the analysis, we found that the "starting point" of the OVH curve for each patient, that was, the value of d when OVH = 0, was significantly different, so the OVH curves was re-classified.

#### Re-classification of OVH

Determining the spatial relationship between the PTV and OARs based on visual similarity can lead to certain deviations. To more accurately distinguish the three-dimensional spatial relationship between the L-Lung and PTV, we introduced a new classification method based on the OVH in our study. For left breast-conserving breast cancer patients, L-Lung has no intersection with PTV (d < 0), only contiguous and separated (d > 0). By expanding the PTV 5 mm (d = 5 mm) uniformly, when

2 $$ {\text{OVH}}\left( {{\text{d}} = {\text{5 mm}}} \right) = \frac{{{\text{V(d}} = {\text{5 mm, L - Lung)}}}}{{\text{V(All, L - Lung)}}}*100\% $$

**Figure 4:**
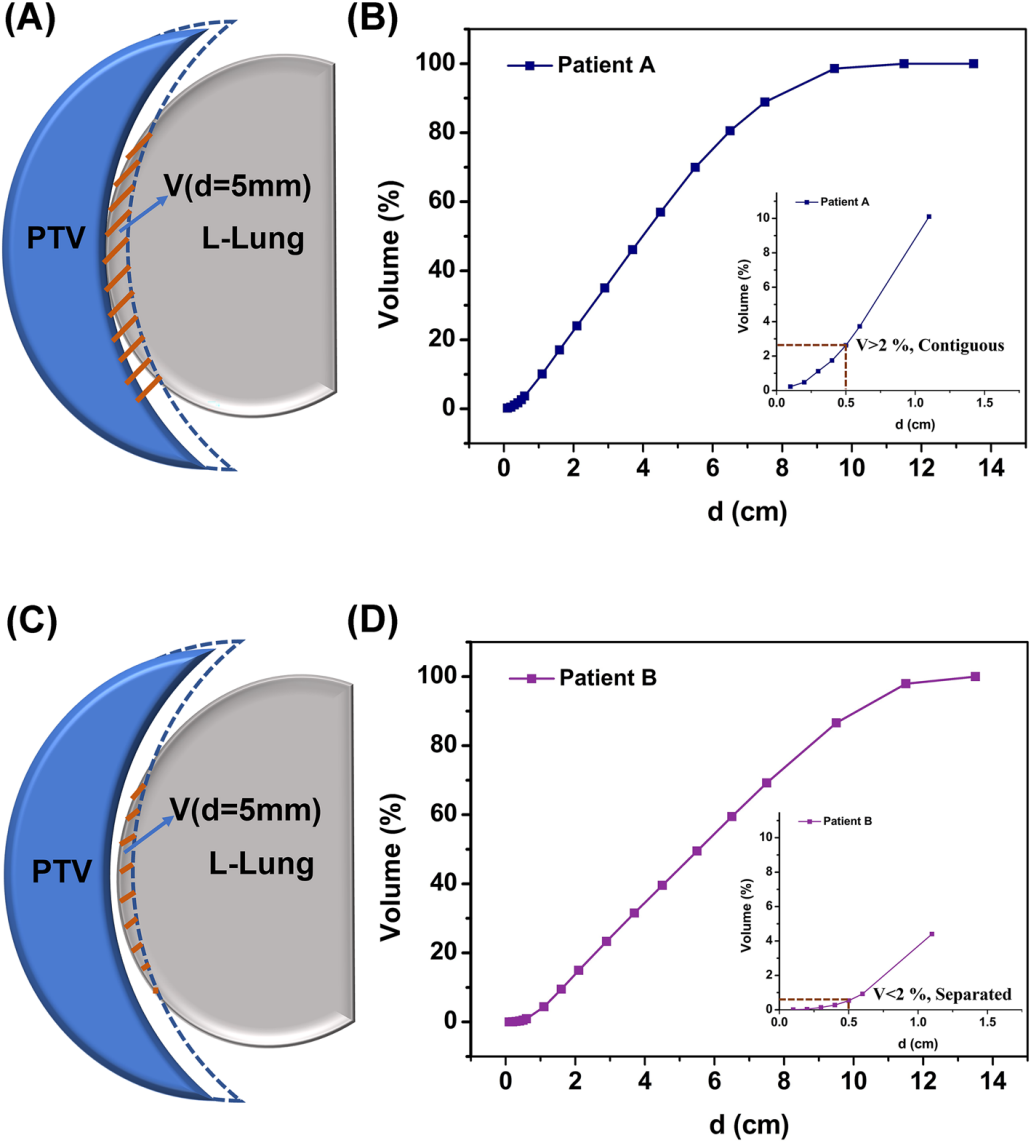
Re-classification of OVH. (**A**, **B**) when OVH(d = 5 mm) > 2% was defined as contiguous; (**C**, **D**) when OVH(d = 5 mm) < 2% was defined as separated.

 > 2% was defined as contiguous (Fig. 4A,B) when OVH(d = 5 mm) < 2% was defined as separated (Fig. 4C,D).

#### The definition and mechanism of trigger (T) operator guiding Radiotherapy plan optimization

According to the above re-classification of OVH, we divided 16 patients into two groups, including a contiguous group (CG, n = 10) and a separated group (SG, n = 6). Subsequently, a trigger (T) operator was introduced to trigger the re-optimization of the plan, the process was as follows:

(a) The volume of V20 (the volume covered by 20 Gy dose line) and the target volume in the 16 original plans were used to calculate the d_n_ (n = 1,2,3,……,16) value, we have

3 $$d_{n} = \sqrt[3]{{V_{20} }} - \sqrt[3]{{V_{PTV} }}$$

(b) Then calculate respectively the $\overline{d }$ values for the CG and the SG, we have

4 $$\overline{d } = \frac{{\sum d_{n} }}{n}$$

then $\stackrel{\mathrm{-}}{\text{d}}$_(CG)_ = 3.3 cm, $\stackrel{\mathrm{-}}{\text{d}}$_(SG)_ = 3.0 cm;

(c) The V value corresponding to the patient's $\overline{d }$ and the V20 value of the L-Lung in the two groups were counted, and the ratio of the two groups was calculated as the trigger (T) operator, we have

5 $$T = \frac{{V\left( {20, L - Lung} \right)}}{{OVH\left( {\overline{d}} \right)}}$$

(d) In formula (5), the smaller the V20, the smaller the T, this plan was considered to be the optimal plan, therefore, select the minimum T value (T_min_) in the CG and SG, and use T_min_ as the trigger (T) operator to trigger the re-optimization of other patient plans respectively (Table 3).

**Table 3:** The value of V20 of the L-Lung and T in the 16 optimal plans in the CG and SG.

CG	Patient NO	1	2	3	4	6	7	8	12	13	14
	V(d = 3.3 cm)	37.00	40.62	31.81	34.85	35.49	37.59	32.6	36.95	38.4	33.46
	V20	15.6	14.5	13.4	11.6	17.2	16.0	17.3	18.4	11.4	11.9
	T_R_	0.42	0.37	0.42	0.33	0.48	0.43	0.53	0.5	0.3	0.36
SG	Patient NO	5	9	10	11	15	16				
	V(d = 3 cm)	28.42	27.47	27.54	30.53	33	31.45				
	V20	11.9	10.5	11.4	14.8	11.9	11.0				
	T_R_	0.42	0.38	0.41	0.48	0.36	0.35				

*Patient NO is the number of 16 patients.

The method of triggering plan re-optimization by T_min_ is the same for CG and SG, taking CG as an example. According to Table 3, T_min, R_ = 0.3 was used as the trigger (T) operator to trigger the first round (R1) re-optimization of the plans of the remaining 9 patients. In the whole optimization process, kept coverage of PTV ≥ 95%, the exposure dose to OARs being below the tolerance dose. Comparing the V20 of the L-Lung in the original plan and the R1 re-optimization plan, it could be concluded that the V20 of the L-Lung in the R1 re-optimization plan was lower than that in the original plan. Calculated again the T value after the R1 re-optimization, T_min, R1_ was used as trigger (T) operators for the second round (R2) re-optimization. After the R2 re-optimization, the re-optimization was terminated in the research.

#### Calculation of radiobiological parameters

According to the Lyman-Kutcher-Burman model, NTCP for Vx of an OAR is given by^9^:

6 $${\text{NTCP}} = \frac{1}{{\sqrt {2\pi } }}\mathop \smallint \limits_{ - \infty }^{t} e^{{ - \frac{{x^{2} }}{2}}} {\text{d}}x$$

where,

7 $${\text{t}} = \frac{{{\text{V}}20 - {\text{V}}20,50}}{{{\text{mV}}20, 50}}$$

where, V20 represents the volume of the OAR receiving the dose of ≥ 20 Gy, and V20, 50 represents the volume received under ≥ 20 Gy when the probability of radiotherapy complications is 50%. In formula (7), the value of m is 0.50, the value of V20, 50 is 30.6.

In addition, according to literature^10^ research results, the probability of radioactive cardiac events will increase by 7.4% when the average cardiac dose increases by 1 Gy, that is:

8 $$\Delta NTCP_{cardiac} = \frac{\Delta MHD(cGy)}{{100cGy}} \times 7.4\%$$

where, the ∆NTCP_*cardiac*_ refers to the change in the probability of cardiac complications before and after optimization, and the ∆MHD refers to the change in the mean heart dose before and after optimization.

In this paper, our team selected formulas (6) and (8) to calculate the NTCP of the L- Lung and heart respectively.

### Statistical analysis

Data are represented as the mean ± standard deviation (SD). Data analysis was performed using GraphPad Prism 5.0. Significance was assessed with a Pairwise Sample T test (*P < 0.05, **P < 0.01). P value of < 0.05 was considered statistically significant.

## Results

### The mechanism of T operator guiding radiotherapy plan optimization

Table 4 shows the mechanism of T operator guiding radiotherapy plan optimization of the patient. The T operator was obtained from the formula (5), and then the T_min_ was selected as the T operator to guide the radiotherapy plan re-optimization. As shown in Table 4, two rounds of optimization could be performed for patients in both the CG and SG. If the V20 of L-Lung obtained after the R2 re-optimization was higher than that obtained after the R1 re-optimization, the R2 re-optimization was considered invalid, and the results of the R1 re-optimization were used for subsequent analysis.

**Table 4:** The mechanism of T operator guiding radiotherapy plan optimization.

CG	Patient NO	1	2	3	4	6	7	8	12	13	14
	V(d = 3.3 cm)	37	40.62	31.81	34.85	35.49	37.59	32.6	36.95	38.4	33.46
	V20	15.6	14.5	13.4	11.6	17.2	16	17.3	18.4	11.4	11.9
	T_R_	0.42	0.37	0.42	0.33	0.48	0.43	0.53	0.5	0.3	0.36
	V20(R1)	14	12.4	9	/	12.9	11.4	11.7	12.2	**—**	10.4
	T_R1_	0.38	0.31	0.28	0.35	0.36	0.3	0.36	0.33	0.3	0.31
	V20(R2)	12.4	11.1	**—**	/	12.6	11.4	/	11.4	10.6	9.6
SG	NO	5	9	10	11	15	16				
	V(d = 3 cm)	28.42	27.47	27.54	30.53	33	31.45				
	V20	11.9	10.5	11.4	14.8	11.9	11				
	T_R_	0.42	0.38	0.41	0.48	0.36	0.35				
	V20(R1)	7.4	5.7	6.5	7.6	8.2	**—**				
	T_R1_	0.26	0.21	0.24	0.25	0.25	0.24				
	V20(R2)	7.3	**—**	6.3	/	/	7.6				

*R1: first round, R2: second round, **—**: indicates that the T-value is used as a trigger operator, and the plan was not re-optimized in this round; /: indicates that the V20 value increases after re-optimization and is not included in the statistics.

### An example of trigger process and results

In this study, we used 16 cases with a prescribed PTV of 50 Gy. All indices of the original plans for these 16 cases met the clinical requirements. Patient no. 12 in the CG was selected as a presentative case to illustrate the principle of V20 of L-Lung re-optimization (Table 4). The V20 of L-Lung was 18.4% in the original plan, and it corresponded to a value of 0.5, which was greater than the minimum value within the group of 0.3, so the plan was triggered for re-optimization. And the V20 significantly dropped to12.2% after the first round of optimization. At this point, its corresponding T value was less than the T value of Patient No.3, which was triggered and optimized again, so it was still the object that was triggered for the second round. And after the second round of optimization, the V20 of Patient no. 12 fell to 11.4%.

### Replanning results of the contiguous and separated groups

**Figure 5:**
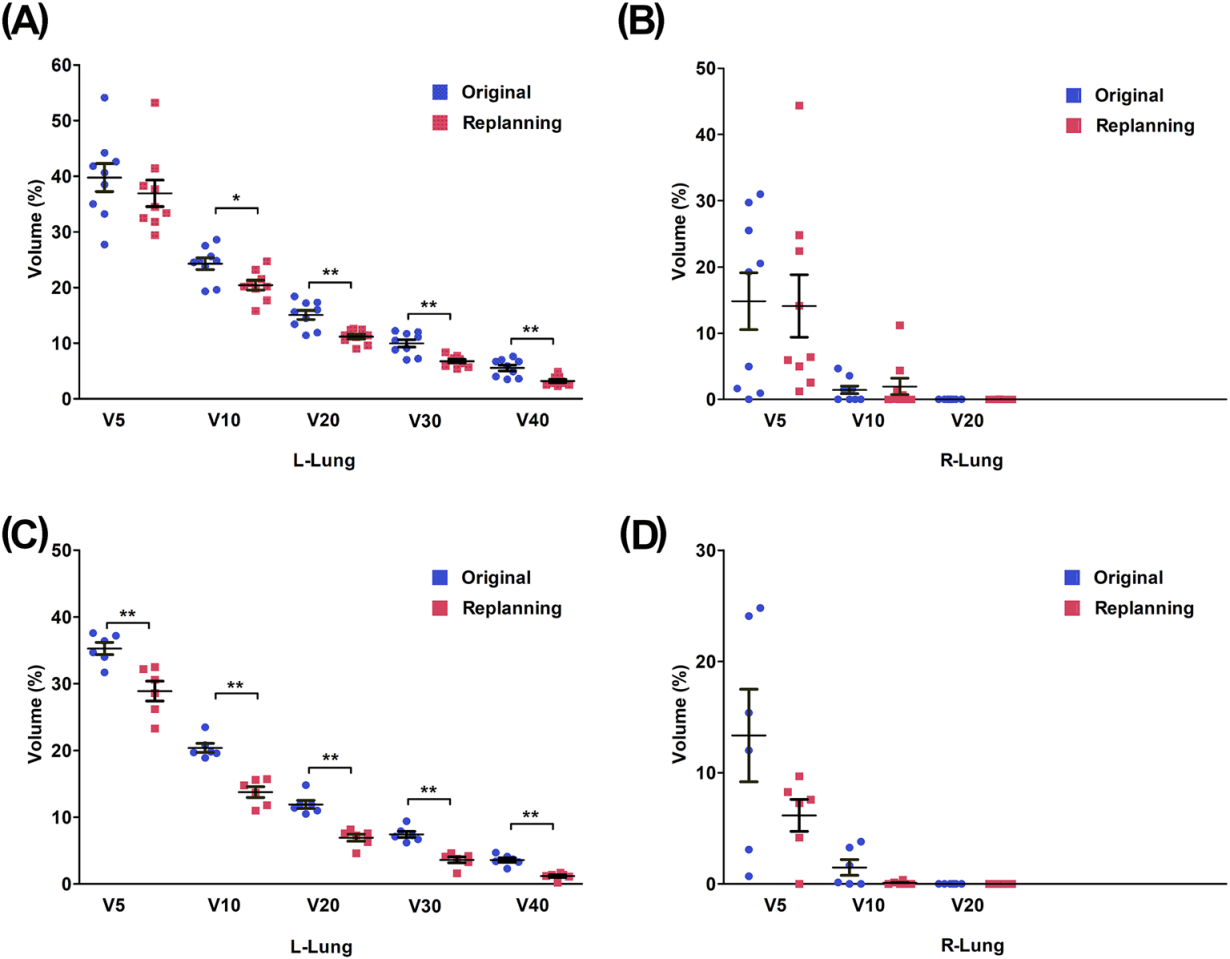
The P values and averages of the Lung of the selected relevant dose/volume points for the original and replanning results. (**A**, **B**) The results of L-Lung and R-Lung in CG; (**C**, **D**) The results of L-Lung and R-Lung in SG; *P < 0.05, **P < 0.01.

Comparing the values of these trigger (T) operator in the original plans, we preliminarily judged that there was a possibility of further decrease in V20 of the L-Lung in 9 cases in the CG and 5 cases in the SG. The Vx of L-Lung and R-Lung were compared between the original plan and re-planning of the 10 cases in the CG and 6 cases in the SG by using the Pairwise Sample T test. The comparisons were illustrated in Fig. 5, the statistical significance was *P* < 0.05 for V10, *P* < 0.01 for V20, V30, and V40 in the CG (Fig. 5A). In addition, all of the 6 patients (n = 6) could also receive lower doses than they did (Fig. 5C). The statistical significance was *P* < 0.01 for V5, V10, V20, V30, and V40 in the SG. The values of V10 and V20 in the R-Lung were almost zero (Fig. 5B,D).

**Figure 6:**
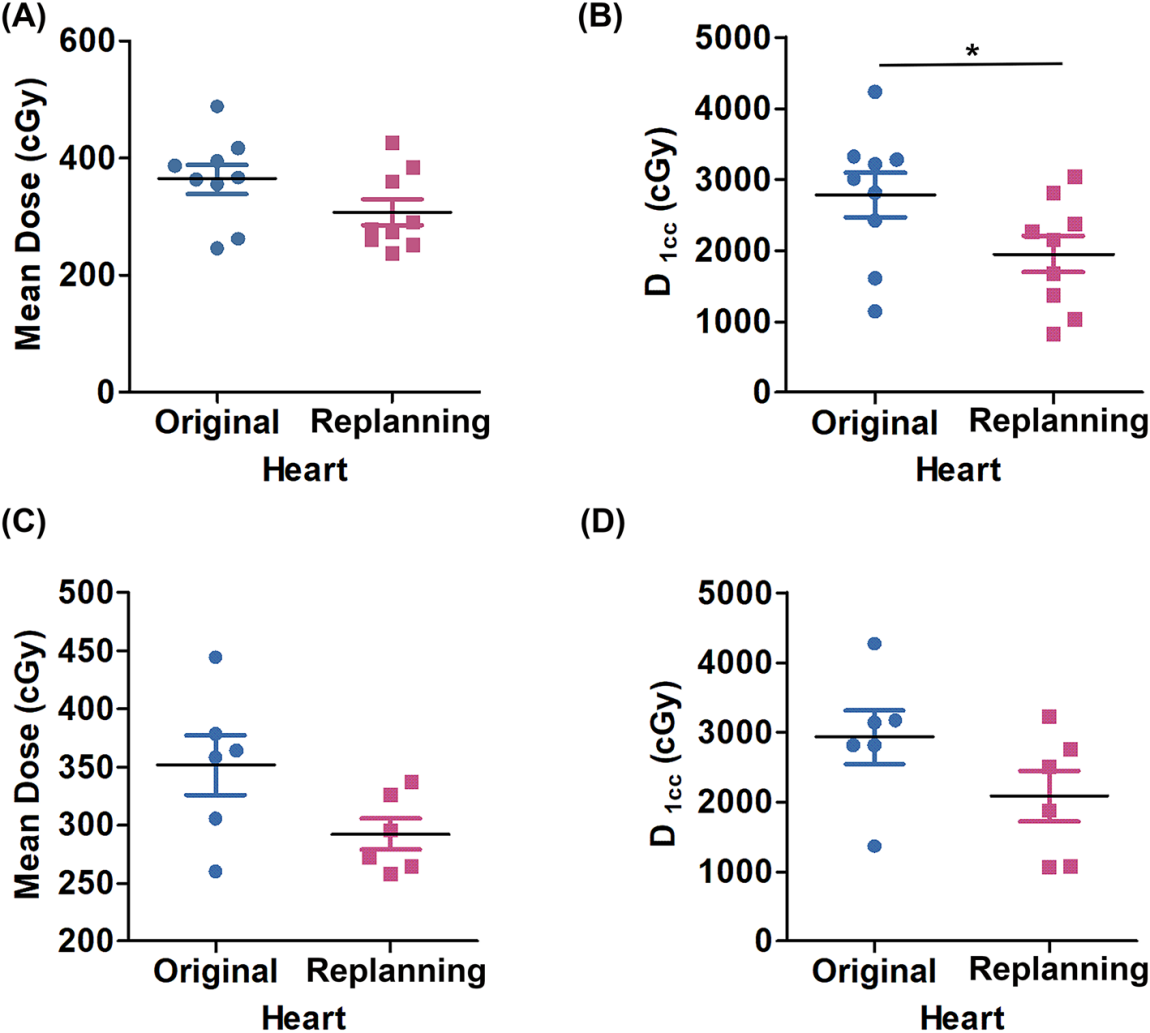
(**A**, **B**) The results of cardiac dose in the CG; (**C**, **D**) The results of cardiac dose in the SG.

Breast cancer radiotherapy often involves some incidental exposure of the heart to ionizing radiation. The study reported the mean dose of cardiac radiation for women with tumors in their left breast was strongly associated with cardiac complications^10^. In our study, we found that after re-optimization, as the dose to the L-Lung decreased after re-optimization, so did the heart (Table 5). The mean and 1 cc volume dose (D_1cc_) of the heart were decreased significantly compared to the original plan after re-optimization in both the CG (Fig. 6A,B) and the SG (Fig. 6C,D).

**Table 5:** Replanning result of the OARs of patient.

Evaluation parameters			Original	Replanning	P value
Heart	CG	MHD (cGy)	364.02 ± 74.28	306.01 ± 66.29	0.052
		D_1cc_ (cGy)	2781.02 ± 940.66	1953.31 ± 770.51	0.016
	SG	MHD (cGy)	351.83 ± 63.40	292.48 ± 33.31	0.087
		D_1cc_ (cGy)	2929.73 ± 935.89	2087.03 ± 895.95	0.060
Breast-R	CG	D_2cc_ (cGy)	1384.94 ± 588.64	1845.83 ± 724.82	0.148
		MBD (cGy)	375.22 ± 171.33	415.28 ± 143.33	0.534
	SG	D_2cc_ (cGy)	1262.30 ± 243.08	2143.81 ± 979.02	0.058
		MBD (cGy)	336.95 ± 118.54	331.18 ± 73.17	0.923
Cord	CG	D_Max_ (cGy)	334.18 ± 155.85	416.22 ± 139.59	0.185
	SG	D_Max_ (cGy)	314.63 ± 97.55	351.61 ± 137.18	0.683

As shown in Table 5, for the contralateral breast and cord, the maximum dose (D_2cc_ in the contralateral breast) and the mean dose increased after re-optimization compared with the original plan.

### Radiobiological results for lung and heart

The results of NTCP of L-Lung were presented in Table 6, the difference in NTCP value of V20 before and after re-optimization was statistically significant (*P* < 0.01 for CG, *P* < 0.001 for SG).

**Table 6:** Comparison of NTCP of L-Lung.

Parameters	Groups	NTCP (Original)	NTCP (Replanning)	t	P
V20	CG	15.79 ± 3.55	10.28 ± 1.36	6.94	0.001
	SG	11.19 ± 1.84	6.15 ± 0.89	5.73	< 0.001

A previous study noted that the risk of a major coronary event increased linearly with the mean dose to the heart. The magnitude of the risk was 7.4% per gray, with no apparent threshold below which there was no risk^10^. The magnitude of the risk value was calculated and shown in Table 7, the incidence of major coronary decreased from 2.69% to 2.27% in the GC (*P* = 0.052) and from 2.60% to 2.16% in the SG (*P* = 0.087).

**Table 7:** Percent increase in rate of major coronary events.

Type	Groups	Original (%)	Replanning (%)	t	P
Heart	CG	2.69 ± 0.52	2.27 ± 0.46	2.279	0.052
	SG	2.60 ± 0.43	2.16 ± 0.23	2.12	0.087

## Discussions

The design of a radiotherapy plan is a critical aspect of radiotherapy, as the quality of the plan determines both the effectiveness of the treatment and the extent of side effects. As a result, many researchers have focused their efforts on improving the quality of radiotherapy plans^11,12^. Among these efforts, one of the most significant contribution has been the advancement in delivery technology. With the introduction of CT and computer technology, 3D-CRT technology replaced 2D-RT technology, and 3D-CRT technology was planned to significantly reduce the dose of peripheral OARs by keeping the shape of the radiation field consistent with the tumor in the BEV direction. Wilson’ s study demonstrated that the percentage volume of whole lung receiving ≥ 20 Gy (V20) was greater in 16 of the 24 2D plans with a median reduction in V20 of 2.4% for 3D (*P* = 0.03)^13^. The intensity-modulated technique has a strong ability to adjust the flux and can be effectively applied to adjust the dose distribution of OARs, effectively reducing OARs’ indicators resulting in reducing the incidence of radiation toxicity. Christian, et al. have shown that IMRT plans could reduce the dose to the lungs compared with 3D-CRT^14^. Over 60 years, intensity modulation technology has evolved, giving rise to various branch technologies, such as static intensity modulation technology (step and shoot), dynamic intensity modulation technology (sliding window), volume modulation arc therapy technology, tomotherapy technology, etc. Jiang, et al.’s study of 12 locally advanced lung cancer patients demonstrated that the target dose coverage of VMAT was better than that of IMRT. For the total and contralateral lungs, the higher V5/10, lower V20/30, and mean lung dose (MLD) were observed in the VMAT plans (*P* < 0.05, respectively). The VMAT technique improved the dose sparing (V20, V30, and MLD) of the control-lateral lung more notably, compared to those parameters of the IMRT^15^. Zhang, et al. compared the efficacy of TomoDirect (TD), Helical Tomat (HT), VMAT, and FF-IMRT for the treatment of esophageal cancer. On the premise that the physical indexes of the target volume meet the clinical requirements, there is no significant difference for the V5, V10, V15, V30, and the mean lung dose (MLD) among the 4 techniques (all *P* > 0.05). However, the V20 differed significantly among TD, HT, VMAT, and ff-IMRT (21.50 ± 7.20%, 19.50 ± 5.55%, 17.65 ± 5.45%, and 16.35 ± 5.70%, respectively; *P* = 0.047)^16^.

However, the challenge of consistently improving plan quality when the technology is fixed remains. To address this issue, a new concept called OVH has been proposed. The OVH calculates a new patient’s DVH by comparing the distances between the OARs and targets of the new patient with those of prior patients whose plans were stored in a database. The basis for this calculation was $r_{v,1} \ge r_{v,2} \Rightarrow D_{v,1} \le D_{v,2}$. For $v > OVH(0)$, we have $r_{v,2} > r_{v,1}$; then $D_{v,2} < D_{v,1}$ was expected. For $v < OVH(0)$, we have $r_{v,1} > r_{v,2}$; then $D_{v,1} < D_{v,2}$ was expected. For $v = OVH(0)$, we have $r_{v,1} = r_{v,2}$; then $D_{v,1} = D_{v,2}$ was expected. This method is effective in predicting the dose of the parotid gland in NPC radiotherapy^7^. However, one shortcoming of this method is that the distance r exists between two points, but the edge of the target in three-dimensional space is a curved surface. Several subsequent studies based on OVH expanded the connotation and application scope of OVH, but the basis of its research was still distance-based^8,17–19^. Early studies on the relationship between distance and dose in breast cancer radiotherapy were pioneered at Oxford University, and the results have been well applied in two/three-dimensional radiotherapy^20^.

It is well known that dose decrement from the target happens in a three-dimensional space, so only considering the distance in the plane is not comprehensive. In other words, a formula $r_{v,1} \ge r_{v,2} \Rightarrow D_{v,1} \le D_{v,2}$, which is not set up in many cases. Therefore, in this study, volume-opening-square ${\text{d}}_{\text{n}}\text{=}\sqrt[{3}]{{\text{V}}_{20}}-\sqrt[{3}]{{\text{V}}_{\text{PTV}}}$ was used to define the distance, and based on this base a trigger operator $\text{T} = \frac{{\text{V}}\left(\text{20, L-Lung}\right)}{{\text{OVH}}\left(\stackrel{\mathrm{-}}{\text{d}}\right)}$, which essentially reflects the dose gradient in the considered space.

The lower the T value is, the higher the dose gradient will be, and the less dose will "diffuse" from the target to the peripheral OARs, which is consistent with the aim pursued by us. So, we chose T_min_ in the cogroup to trigger the re-optimization of the other plans. The reduction of T-value is essentially a process of dose concentration to the target, and one of the results of this process is a decrease in the dose which diffused into around OARs from targets, which is manifested as a decrease in OARs ' Vx. This is why 14 patients in Table 4 were triggered, optimized, T value and V20 decreased.

Of the 10 original plans in the CG, the T value of Patient NO.13 was the minimum, and the T value of other patients was higher than 0.3, which indicated that the dose gradient of their plans could be increased further. So, in the re-optimization, the planner added a function to the cost functions to increase the dose gradient. After the R1 re-optimization, a value lower than 0.3, 0.28, appeared, thus triggering the R2 re-optimization. After the R2 re-optimization, in some patients, the Vx in the L-Lung was further reduced, while in others it was not. The reason is that the dose drop is also affected by the shape of the target and the position of the target relative to other OARs. It can be seen that the selected T_min_ for each round of optimization is not constant. Because the selected T_min_ for each round is only a relatively small value, it is not necessarily the minimum value that can be achieved. In the CG group in Table 4, the trigger operator T_min_ = 0.3 in the first round of trigger optimization is a relatively small value, which triggers other patient protocols to be optimized. However, after the first round, another relatively small value of 0.28 appeared, so the original T = 0.3 corresponding to the plan became the object that was triggered to optimize. But it is foreseeable that there will be no sustained relatively small value, because there is a limit to how much the dose can fall.

In theory, the T_R_ cutoff (the minimum value that can be achieved) should have a definitive value, which corresponds to the limit of dose drop. However, in clinical practice, the experience and expertise of the designers play a crucial role in determining the value of the T_R_ cutoff. In our study, we conducted two rounds of triggers and optimization, and despite optimizing the third round for all enrolled plans, we did not achieve a lower V20 in any case. Therefore, our team only conducted two rounds of triggers in this study.

In this study, the enrolled patients were divided into two groups. The reason was that the most important OAR was L-Lung in this study. However, the distance between L-Lung and the target varied greatly among patients. Some patients had the target adjacent to the L-Lung, while others had a thick chest wall between the target and the L-Lung. However, the chest wall was not considered as an OAR in this study. For ‘having-thick-chest-wall’ patients, when planners adjusted the dose, the dose gradient variance was not only in L-Lung but also in the thick chest wall. Hence, dividing the patients into two groups was necessary.

The purpose of reducing the physical dose of OAR is to decrease NTCP, and the prediction of NTCP is a complex problem, mainly due to variations in individual patient responses to radiation^21,22^ and the unique characteristics of different OARs^23,24^. Using a mathematical formula to calculate NTCP is not comprehensive^25,26^, but it is currently an international practice because it can solve most questions within a certain range. In this study, L-Lung NTCP is calculated based on V20, because (1) V20 is the most important physical dose index affecting lung NTCP; (2) The main objective of the re-optimization in this study is to reduce the V20 of L-Lung. One of the coincidences in this study is that the L-Lung is on the same side of the tumor as the heart, so the dose "diffused" to the L-Lung went down at the same time as the dose "diffused" to the heart went down. Unlike lung NTCP, heart NTCP is proportional to the mean dose of the heart. Therefore, the research team used different formulas based on different physical doses to calculate the change in the probability of complications for L-lung and Heart, formulas (6) and (8). The results in Table 6 and 7 showed that the decrease in dose led to a decrease in the probability of complications, which indirectly proved the potential clinical value of the proposed method in this paper.

The purpose of this paper is to recommend a method for improving plan quality. It is well known that continuously improving the quality of plans and keeping the quality level of plans from declining due to the different experiences of the physicist (dosimetrist) are important tasks for each physicist (dosimetrist) team. Through the trigger proposed in this paper, we can review the team's previous breast-conserving radiotherapy plans to obtain a trigger value to guide the design of new plans or extract a trigger value from the plans designed by experienced physicists (dosimetrists) to guide the plan design of entry-level physicists (dosimetrists). This is one of the practical implications of this paper. Another potential implication is to programmatically build the recommended methods into TPS to evaluate plans or drive automatic plan optimization.

## Conclusion

The proposed trigger operator could enhance plan quality for reducing radiation-induced lung injury in breast cancer postoperative radiotherapy.

## Data Availability

The datasets analyzed during the current study are available from the corresponding author on reasonable request.

## Abbreviations

OVH: Overlap volume histogram
VMAT: Volumetric modulated arc therapy
CG: Contiguous group
SG: Separated group
OARs: Organ-at-risks
NTCP: Normal tissue complication probability
IMRT: Intensity modulated radiation therapy
DVH: Dose volume histogram
PTV: Plan target volume
CT: Compute technology
T: Trigger
SD: Standard deviation
MLD: Mean lung dose
TD: Tomo Direct
HT: Helical Tomat

## References

[1] Villaggi E (2019). Plan quality improvement by DVH sharing and planner's experience: Results of a SBRT multicentric planning study on prostate. Phys. Med..

[2] Kubo K (2019). Inter-planner variation in treatment-plan quality of plans created with a knowledge-based treatment planning system. Phys. Med..

[3] Oetzel D (1995). Estimation of pneumonitis risk in three-dimensional treatment planning using dose-volume histogram analysis. Int. J. Radiat. Oncol. Biol. Phys..

[4] Graham MV (1999). Clinical dose-volume histogram analysis for pneumonitis after 3D treatment for non-small cell lung cancer (NSCLC). Int. J. Radiat. Oncol. Biol. Phys..

[5] Zhagn Z (2014). Risk factors of radiation-induced acute esophagitis in non-small cell lung cancer patients treated with concomitant chemoradiotherapy. Radiat. Oncol..

[6] Kim SW (2020). Loco-regional outcomes of adjusted breast radiotherapy with conventional fractionation after breast conserving surgery: De-escalation of whole breast irradiation dose. Medicine.

[7] Wu B (2009). Patient geometry-driven information retrieval for IMRT treatment plan quality control. Med. Phys..

[8] Zhou Z, Zhang W, Guan S (2016). An effective calculation method for an overlap volume histogram descriptor and its application in IMRT plan retrieval. Phys. Med..

[9] Borst GR (2010). Radiation pneumonitis after hypofractionated radiotherapy: evaluation of the LQ(L) model and different dose parameters. Int. J. Radiat. Oncol. Biol. Phys..

[10] Darby SC (2013). Risk of ischemic heart disease in women after radiotherapy for breast cancer. New England J. Med..

[11] Ahmad I (2020). Plan quality assessment of modern radiotherapy delivery techniques in left-sided breast cancer: an analysis stratified by target delineation guidelines. BJR Open.

[12] Lymberiou T (2015). Predictors of breast radiotherapy plan modifications: quality assurance rounds in a large cancer centre. Radiother. Oncol..

[13] Wilson EM (2005). Comparison of two dimensional and three dimensional radiotherapy treatment planning in locally advanced non-small cell lung cancer treated with continuous hyperfractionated accelerated radiotherapy weekend less. Radiother. Oncol..

[14] Chun SG (2017). Impact of intensity-modulated radiation therapy technique for locally advanced non-small-cell lung cancer: a secondary analysis of the NRG oncology RTOG 0617 randomized clinical trial. J. Clin. Oncol..

[15] Jiang X (2011). Planning analysis for locally advanced lung cancer: dosimetric and efficiency comparisons between intensity-modulated radiotherapy (IMRT), single-arc/partial-arc volumetric modulated arc therapy (SA/PA-VMAT). Radiat. Oncol..

[16] Zhang Y (2019). Dosimetric comparison of TomoDirect, helical tomotherapy, VMAT, and ff-IMRT for upper thoracic esophageal carcinoma. Med. Dosim..

[17] Wu B (2014). Improved robotic stereotactic body radiation therapy plan quality and planning efficacy for organ-confined prostate cancer utilizing overlap-volume histogram-driven planning methodology. Radiother. Oncol..

[18] Wu B (2013). Using overlap volume histogram and IMRT plan data to guide and automate VMAT planning: a head-and-neck case study. Med. Phys..

[19] Yang Y (2013). An overlap-volume-histogram based method for rectal dose prediction and automated treatment planning in the external beam prostate radiotherapy following hydrogel injection. Med. Phys..

[20] Taylor CW (2009). Estimating cardiac exposure from breast cancer radiotherapy in clinical practice. Int. J. Radiat. Oncol. Biol. Phys..

[21] Xu SH (2022). Osteoradionecrosis of the hip, a troublesome complication of radiation therapy: case series and systematic review. Front. Med..

[22] Komaei I (2019). Radiation-induced undifferentiated pleomorphic sarcoma of the breast: a rare but serious complication following breast-conserving therapy. A case report and literature review. Il Giornale di chirurgia.

[23] Fukada J (2021). Mean heart dose-based normal tissue complication probability model for pericardial effusion: a study in oesophageal cancer patients. Sci. Rep..

[24] Bai H (2020). Test the effectiveness of quantitative linear-quadratic-based (qLQB) model on evaluating irradiation-induced liver injury (ILI) against normal tissue complication probability (NTCP). Dose-Response.

[25] Palma G (2019). Normal tissue complication probability (NTCP) models for modern radiation therapy. Seminars Oncol..

[26] Brodin NP (2018). Systematic review of normal tissue complication models relevant to standard fractionation radiation therapy of the head and neck region published after the QUANTEC reports. Int. J. Radiat. Oncol. Biol. Phys..

